# Community engagement and mobilisation of local resources to support integrated Community Case Management of childhood illnesses in Niger State, Nigeria

**DOI:** 10.7189/jogh.09.010804

**Published:** 2019-06

**Authors:** Ayodele Alegbeleye, John Dada, Olusola Oresanya, Jonathan Jiya, Helen Counihan, Patrick Gimba, Lynda Ozor, Kolawole Maxwell

**Affiliations:** 1 Malaria Consortium, Minna, Niger state, Nigeria; 2 Malaria Consortium, Abuja, Nigeria; 3 Malaria Consortium, London, UK; 4 Niger State Ministry of Health, Minna, Nigeria; 5 World Health Organization, Abuja, Nigeria

## Abstract

**Background:**

Despite strong evidence of integrated community case management (iCCM) of childhood illnesses being a proven intervention for reducing childhood morbidity and mortality, sustainability remains a challenge in most settings. Community ownership and contribution are important factors in sustainability. The purpose of this study was to document the process and scale achieved for community engagement and mobilisation to foster ownership, service uptake and sustainability of iCCM activities.

**Methods:**

A review of data collected by the RAcE project was conducted to describe the scale and achievement of leveraging community resources to support the community-oriented resource persons (CORPs). The Rapid Access Expansion (RAcE)-supported iCCM programme in Niger state (2014-2017), aimed at improving coverage of case management services for malaria, pneumonia, and diarrhoea, among children aged 2–59 months. Resources donated were documented and costed based on the market value of goods and services at the time of donation. These monetary valuations were validated at community dialogue meetings. Descriptive statistics were used to summarise quantitative variables. The mean of the number of CORPs in active service and the percentages of the mobilised resources received by CORPs were calculated.

**Results:**

The community engagement activities included 143 engagement and advocacy visits, and meetings, 300 community dialogues, reactivation of 60 ward development committees, and 3000 radio messages in support of iCCM. 79.5% of 1659 trained CORPs were still in active iCCM service at the end of the project. We estimated the costs of all support provided by the community to CORPs in cash and kind as US$ 123 062. Types of support included cash; building materials; farming support; fuel for motorcycles, and transport fares.

**Conclusions:**

The achievements of community engagement, mobilisation, and the resources leveraged, demonstrated acceptability of the project to the beneficiaries and their willingness to contribute to uninterrupted service provision by CORPs.

In 2016, mortality among children under-five years (Cu5) in Nigeria was estimated at 104 deaths per 1000 live births [1]. Pneumonia, diarrhoea and malaria account for 32 percent, 16 percent and 19 percent of deaths in Cu5 in Nigeria [2]. Cu5 are more vulnerable to these diseases than other segments of the population, and can die without access to treatment, especially in rural areas, where access to the nearest health centre can be difficult. Mortality rates in rural areas are consistently higher than in urban areas linked to a relative dearth of human resources for health in those hard-to-reach areas [3]. In response to high morbidity and mortality among Cu5, WHO and UNICEF recommend integrated community case management [iCCM] as a strategy to provide timely and effective treatment of the three focal diseases: malaria, pneumonia and diarrhoea among Cu5 [4]. iCCM is proven to be effective in increasing treatment coverage and delivering quality care for sick children, especially in areas with limited access to facility-based health care providers [4-7].

The service delivery for iCCM typically depends on lay, often volunteer, community health workers (CHWs) selected and trained to render the service in their communities [4]. Successful and sustainable iCCM services depend on appropriate selection, training, supervision and support to these CHWs [8-10]. However, there are challenges related to engagement and sustaining of volunteer health workers. The challenges include inadequate number of volunteers who have a suitable profile to perform as a CHW; inappropriate incentives and compensation for the volunteers; and lack of support from the formal health system and community leadership [11,12]. These challenges have resulted in varied rates of performance and retention among these workers with an attrition range of 3% to 77% documented in a variety of community health delivery programmes [12-14]. Attrition of CHWs negatively affects access to, and utilisation of iCCM services [11,12]; and improvement in utilisation of services has been shown to be dependent on effective demand creation and social mobilisation [8,15].

Approaches used for demand creation and resource mobilisation for iCCM projects implemented previously have been documented. This often involve a three-pronged approach to include social and behaviour change communication, social mobilisation and advocacy [15]. Evidence has shown that social mobilisation is one of the key features of successful public health programmes [16,17]. However, the need to continue to assess and address the demand barriers for iCCM through behaviour change, community engagement and social mobilisation activities was identified at an international symposium in 2014 on lessons and priorities for iCCM [8].

## Community Engagement and Mobilisation for RAcE Project

The Rapid Access Expansion 2015 (RAcE) project was the first iCCM programme at scale in Nigeria. It was implemented in collaboration with state government and partners, led by Malaria Consortium in Niger State, and funded by Global Affairs Canada through WHO. The partnership for the project included state government, the six focal local government areas (LGAs), and Federation of Muslim Women Association of Nigeria (FOMWAN), a faith-based non-governmental organisation (NGO). The goal of the project was to contribute to the reduction in mortality and morbidity among children aged 2 to 59 months. The emphasis was to increase access to correct diagnosis, treatment and referral for malaria, pneumonia and diarrhoea among children – Cu5 at the community level. The components of the RAcE project were service delivery, capacity building, demand creation, and monitoring and evaluation.

A key feature of the RAcE project was the selection and training of volunteers at the community level. The nomination of community members as volunteer CHWs was done by the community leaders, based on the selection criteria provided by the National iCCM Task Team. The main selection criteria were the volunteer’s ability to read and write; and being resident in the communities where they will offer iCCM services. The nominees were trained by state trainers of CHWs, who were themselves trained at training of trainers organised by the state government, with the support of Malaria Consortium. Trainees whose performance at the training passed a required standard were selected as CHWs for iCCM. The trained volunteer CHWs are designated in Nigeria as community resource persons (CORPs), and they provide free iCCM services for the three focus diseases. To support this programme, another set of volunteers called social mobilisers (SMs) were selected in a process similar to that of the CORPs. They were trained to conduct mobilisation of community members to demand for, and to use, iCCM services as well as sensitising them on appropriate health seeking behaviour. The training and deployment of SMs was integral to community mobilisation.

**Figure 1:**
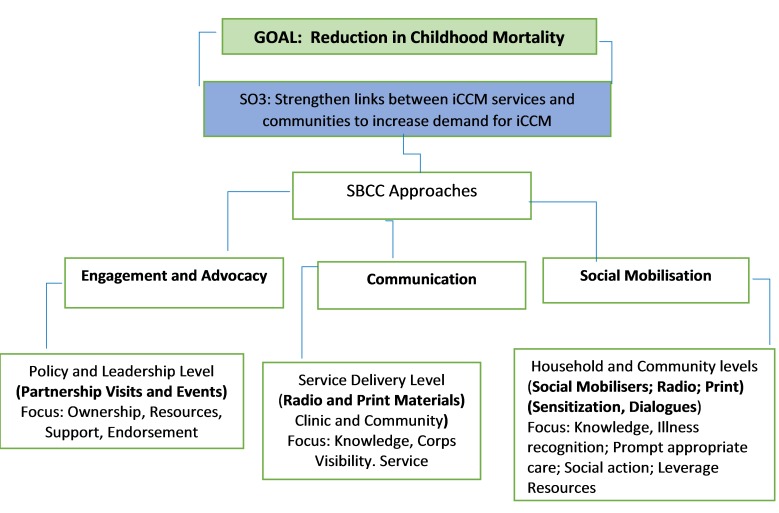
Approaches and Components of Community Engagement and Mobilisation in RAcE project, Niger state. Adapted from Sharkey et al [15].

The engagement and mobilisation component of the RAcE project was developed to strengthen links between iCCM services and the communities, and to engender ownership and sustainability of the project. The three broad communication approaches adopted were engagement and advocacy, communication, and social mobilisation (**Figure 1**), reflecting social and behaviour change communication frameworks from other public health programmes [17-19]. At the onset of the project, policy-level advocacy and engagement meetings were conducted. These included an inception meeting where government and other stakeholders at state and local government area (LGA) levels were briefed on the objectives of the iCCM programme and the processes involved. Other platforms and opportunities were used to continually solicit support and commitment to sustain the gains of the project. An iCCM sustainability roadmap development workshop was organised by the state government and partners, where community leaders were invited to share their perspectives and make suggestions for community buy-in.

Advocacy and engagement activities at the community level were implemented in partnership with Federation of Muslim Women’s Associations in Nigeria (FOMWAN) and other relevant community-based organisations (CBOs) as appropriate, and targeted at the community leaders as gatekeepers of the communities. The other CBOs included representatives of the women’s wing of various Christian churches. FOMWAN and the women’s organisations participated in identification of key advocacy issues, the advocacy visits, and presentations and follow-up actions to address the issues. A total of 143 advocacy visits and meetings were held at the community level with leadership of the communities. These included religious leaders, paramount traditional rulers, district heads and the leadership of faith-based organisations (FBOs) in the six focal LGAs. The iCCM-related advocacy needs and problems amenable to leadership support were presented to the leaders, who worked to promote iCCM. The key advocacy issues and the ‘asks’ included soliciting continuous leadership goodwill and support for iCCM, including their involvement in the selection of the CORPs, and provision of incentives and non-monetary support to the CORPs.

Key communication messages and materials were produced to support mobilisation activities and targeted at the community members. These messages in radio formats (mainly radio spots totalling 3000 slots), public service announcements and discussion programmes were aired to promote the CORPs, and the utilisation of iCCM services. The print formats involved production of flipcharts and motivational leaflets on iCCM, with images and basic information on the three focal diseases. The messages and materials were developed in English, and translated into three local languages.

The social mobilisation activities were conducted at the community level to introduce iCCM, explain its purpose, and benefits, and to and seek involvement and participation of the key audiences at the community level. The primary target were the caregivers and heads of households as community members. One hundred and twenty-six SMs were selected and trained for two days on community engagement and mobilisation approaches including sensitisation meetings, community dialogues, community level advocacy, resource mobilisation and other social mobilisation activities. The primary role of SMs was sensitising community members on appropriate health seeking behaviour, mobilising them to demand for iCCM services, supporting the reactivation and strengthening of community health structures such as village and ward development committees (WDCs) as well as mobilising community level support for CORPs.

The SMs were given data capturing tools to report and document their activities which were monitored and supervised by either the LGA Health Educators or FOMWAN officials trained to provide supervisory support to the SMs. A series of community dialogues (CDs) were conducted in small groups to achieve two purposes: initially to explore issues related to iCCM, and also to identify priority issues and develop follow-up actions to be jointly implemented by community members. Participants at the CDs were the community members, SMs, the CORPs and representatives of FOMWAN, CBOs and FBOs. The facilitators at the CDs were the SMs, and the LGA health educators and staff of Malaria Consortium. The needs of the CORPs and challenges related to their work were discussed during these CD sessions and community meetings, decisions taken on how to address them and follow up actions jointly implemented by community members, SMs and state iCCM team. One of the critical needs identified across all LGAs was the need to support the CORPs with monetary and non-monetary resources to enhance their commitment as volunteer health service providers.

As part of the engagement and mobilisation activities, 60 ward development committees (WDCs) were reactivated in the six focal LGAs. The WDCs were formed as part of the community engagement structure for primary health care programme. Committee members are made of representative of the different sector of development including health, education and agriculture. Their purpose is to work with government and partners to identify and collectively address key development issues at the community level. A summary of community engagement and mobilisation activities is presented in **Table 1**.

**Table 1:** Engagement, audiences, and the outputs

Audiences for engagement	Key outputs
Community members	Participation in the state roadmap workshop on iCCM
Traditional Rulers	CORPs (n=1659) selected and trained for ICCM
Religious leaders	143 advocacy visits conducted
Leadership of faith-based Organisation (FBOs)-WOWICAN, CAN	60 Ward Development Committees reactivated
Leadership of Ward Development Committees	Active participation in mobilisation activities (Regular attendance, motivating self and others, acceptance of roles, and follow-up on action points)
	Utilisation of ICCM services as indicated in the results quantified for the project

iCCM – integrated community case management, CORP – community-orientated resource person

As partners and stakeholders continue to address the challenges around sustainability of iCCM, this study was conducted to document the engagement, and the resources mobilised at the community level to support the CORPs in the RAcE project in Niger state; and as a key aspect of sustainability of iCCM in the state.

## METHODS

This was a descriptive study focused on the scale and achievements of mobilisation of community resources as part of the engagement and community mobilisation component of the RAcE project, primarily by retrospectively reviewing data routinely collected by the project.

### Project setting

**Figure 2:**
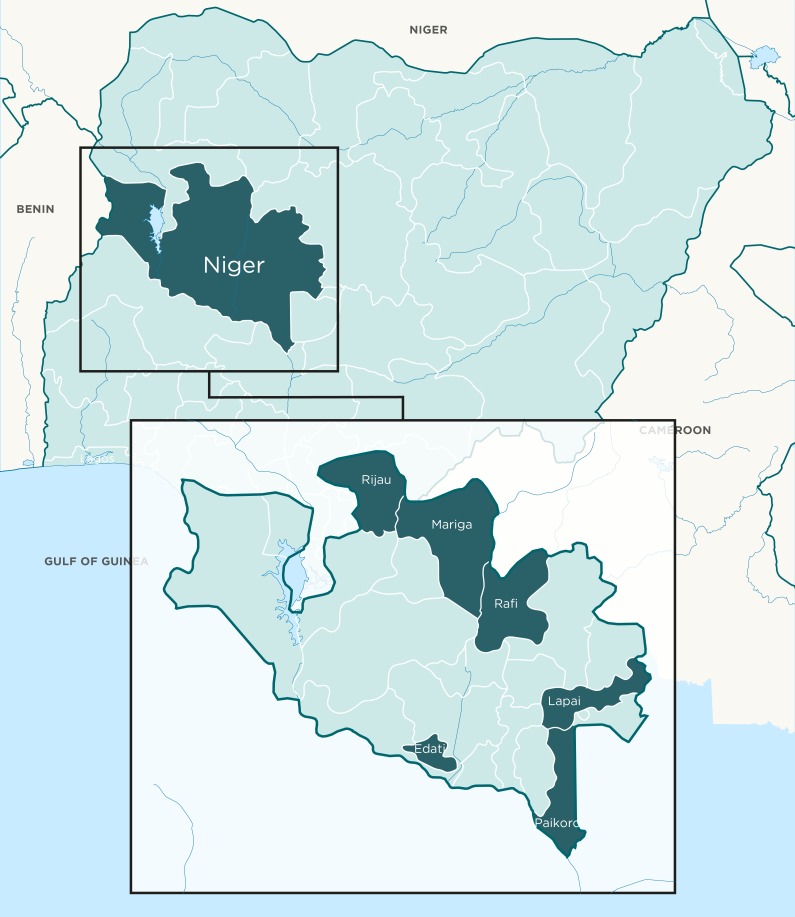
RAcE project local government authority (LGAs) in Niger state.

The RAcE project (2014-2017) was implemented in six local government areas (LGAs) in Niger State, North Central Nigeria (**Figure 2**). The six LGAs: Edati, Lapai, Mariga, Paikoro, Rafi, and Rijau, had a combined population of 1 424 226 as at 2013, based on projections from the 2006 census [20]. Niger state comprises three main ethnic groups (Nupe, Hausa, and Gbagyi) with different religious and cultural backgrounds, and socio-economic status. Most project communities are without potable water and electricity and are located far away from health facilities. Niger State is largely rural and agrarian, with farming and fishing as main occupation; and with strong cultural structures and institutions, where there is high respect for traditional and religious leaders.

A total of 1659 volunteers trained as CORPs made up the study population, with 1320 confirmed to be still in active iCCM service through a validation exercise at the end of the third year of the project. The 1659 CORPs were considered the actual and potential beneficiaries of the engagement and mobilisation activities including resources mobilised from the community.

### Data sources and collection

Mobilised resources as variables were assessed based on the estimation of the cost of these resources donated to support the CORPs and iCCM. Details of these resources, both monetary and non-monetary, and their value were described by community members and entered in a project resource mobilisation form by the SMs, under the guidance of the government staff, and officers of the RAcE project team. Entries on the resource mobilisation form were validated at community dialogue meetings, based on the market value of items at the time of the donation. Donated items and labour were costed based on the prevailing cost of goods and services at the time they were donated. The financial support, and estimated cost of the items donated to the CORPs in each LGA were aggregated for the period of three years, as the value under each of the categories of mobilised resources; and used as the quantitative data for this independent variable.

Community members attending community dialogues, and SMs, contacted representatives of trade and markets associations to determine the current market price of the items, labour, and gifts donated to the CORPs. The entries on the resource mobilisation form were only validated after the cost of the items have been confirmed by representatives of the trade and market associations, following due diligence with the associations. The approach adopted for costing of the items mobilised for the project conforms with the recommendation of how to document in-kind contributions to a project [21]. The category of resource mobilised in support of the CORPs and ICCM services are presented in **Table 2**.

**Table 2:** Categories of monetary and non-monetary contributions by the community to support the CORPs on the RAcE Project

1. Cash.
2. Farming support & chemicals (labour as farming support).
3. Farm produce and animals (goats, ram, sheep, bags of grains).
4. Houses built (Building materials: mud blocks, roofing sheets, cement, sand provided to CORP for building house).
5. Logistics support/aids (Female CORP had firewood and water fetched for them; assisted with labour for laundry).
6. Motorcycle fuel or transport fare to collect drugs from supervising facilities.
7. Support to male CORPs to host marriage ceremonies.
8. Motorcycle and bicycle purchased for CORPs.

CORP – community-orientated resource person, RAcE – Rapid Access Expansion

### Statistical methods

Frequency tables were generated for the resource mobilisation variables assessed. Descriptive statistics such as means were used to summarize quantitative variables while categorical variables were summarised with proportions. The mean of the number of CORPs in active service was calculated. The percentage of the mobilised resources received by CORPs in each LGA was calculated.

## RESULTS

### Monetary and non-monetary community support provided to the CORPs

The mobilised resources in support of the services of the CORPs were grouped into seven categories including monetary, and non-monetary incentives, as shown in **Table 1**. Many were in the form of support for farming and iCCM activities, including purchase of vehicles and support for construction of houses.

Over the period of 3 years (2015-2017), the total resources mobilised in support of the retained CORPs was estimated as the sum of US$ 123, 062 of which 14.8% was monetary (cash) while the remaining 85.2% was in form of non-monetary incentives, including farm labour, building materials and others (**Table 3**). The mobilised resources included farming support and chemicals (55.21%); farm produces and animals (17.5%), financial assistance (4.8%), and support to CORPs for marriage (0.4%). The mobilised resources varied by LGA: Edati (37.7%), Lapai (33.42%) and Paikoro (1.64%).

**Table 3:** Estimated community resources mobilised by recipient CORPs in the six local government areas (LGAs), RAcE Project

		LOCAL GOVERNMENT AREAS							
Type of resources mobilised by number of corps (CORT No.) and estimated monetary value (EMV) in US$*		**Paikoro**	**Rafi**	**Mariga**	**Edati**	**Lapai**	**Rijau**	**Total CORPs**	**Amount of resources mobilized, US$ (%)**
Cash	CORP No	45	54	84	65	104	81	433	
	Direct Cash (US$)	804	3908	1073	5229	6128	988		18 129 (14.8)
Farming Support & Chemicals	CORP No.	15	58	43	69	68	49	302	
	EMV	401	6654	1575	23 987	30 456	4715		67 787 (55.2)
Houses Built/ mud blocks	CORP No.	–	6	2	2	–	2	12	
	EMV	–	4670	843	5348	–	1797		12 657 (10.1)
Logistics Support/Aid	CORP No.	12	8	8	2	3	14	47	
	EMV	253	235	281	17	96	554		1436 (1.2)
Support for Marriage for CORPs	CORP No.	1	4	–	–	1	–	6	
	EMV	63	354	0	0	112	0		530 (0.4)
Motorcycle/ Bicycle purchased for CORPs	CORP No.	1	4	1	-	-	1	7	
	EMV	352	349	240	0	0	73		1014 (0.8)
Farm Produce/ Animals	CORP No.	4	16	69	66	41	105	301	
	EMV	134	740	222	11 778	4243	4391		21 508 (17.5)
Total	EMV	2008 (1.6)	16 911 (13.7)	4234 (3.4)	46 359 (37.7)	41 036 (33.3)	12 479 (10.2)		123 062 (100.0)

CORP – community-orientated resource person, RAcE – Rapid Access Expansion

*Resources estimated in Naira and converted to US$ at exchange rate of U$ 1 = N 312.99; the parallel market rate in the country between 2016 and 2017.

### Retention of CORPs in iCCM

A total of 1659 CORPs were trained, of which 1320 (79.5%) were in active iCCM service at the end of year 3 of the project (**Table 4**). The proportion of the active CORPs was highest in Rijau LGA (90.70%), followed by 88.21% and 77.10% in Lapai and Mariga LGAs respectively; with the lowest in Edati LGA (62.42%).

**Table 4:** Active CORPS, RAcE Project, Niger state

LGAs	Number CORPS trained; Years 1-3	Number and % of CORPs in active iCCM service by end of Year 3	% of CORPs in active iCCM service by end of Year 3 in 6 LGAs
Paikoro	213	159 (74.65)	79.57
Rafi	265	202 (76.23)	
Mariga	393	303(77.10)	
Edati	189	118(62.42)	
Lapai	212	187(88.21)	
Rijau	387	351(90.70)	
**TOTAL**	**1659**	**1320**	

LGA – local government authority, iCCM – integrated community case management, CORP – community-orientated resource person, RAcE – Rapid Access Expansion

### Other observed effects of resource mobilisation to support iCCM

Based on meeting records and anecdotal feedback from the community meetings and from the SMs and project staff, a number of further observations were made about the nature and consequences of this community engagement and mobilisation including resource mobilisation as follows

- All the CORPs, irrespective of their gender, enjoyed recognition in their communities
- The community members showed their appreciation and commonly referred to the CORPs them as “local doctors”, in recognition of their services.
- In Rafi LGA, two communities dedicated common farmland to iCCM from which the harvest will be sold and the money used to fund iCCM activities. A bi-weekly contribution of US$ 0.80 per head was levied to fund iCCM activities at the community level. Some communities have also indicated willingness to sustain procurement of iCCM drugs and commodities after the close-out of the RAcE project.
- Election of two CORPs as Ward Councillors, a position at the lowest level of political administration in the country but one of important standing in the community
- A female CORP in one of the six LGAs was engaged by the LGA Primary Health Care department as staff following her commitment and high-quality support for Cu5 care while her other colleagues enjoyed monthly support of the LGA leadership to collect drugs and other commodities from the supporting health facilities
- The paramount traditional leader of Lapai, in his public support for CORPs, noted that “there is no greater work than providing good health, and there is no greater worker that should be appreciated than the person who is trying to improve your health”.
- A popular religious leader in Lapai LGA, whose child has been a direct beneficiary of the ICCM intervention noted ‘’I advise parents to take their sick children to the CORPs; urged government and communities to support the CORPs”.

## DISCUSSION

### Monetary and non-monetary community support provided to the CORPs

While many studies have indicated that community appreciation and support are important motivators for CHWs [22], the type and scale of community level resources mobilised in support for the RAcE iCCM project have rarely, if ever, been previously documented from other similar CHW programmes in the same level of detail. The generated resources in the form of monetary and non-monetary community level support for the trained CORPs were substantial, considering the bulk were contributed from meagre resources of subsistence farmers, technicians and artisans. Although the community resources mobilised for the CORPs varied by LGA, it was particularly high in two LGAs, Edati and Lapai, where nearly three quarters of the entire resources were mobilised.

It was not a surprise that about four-fifths of the resources mobilised to support the CORPs were non-monetary, just as the finding that over half of the estimated support was in the form of family support and agrochemicals for farming. Only resources considered crucial and most needed by CORPs were agreed at community dialogue sessions. Farming remains the main occupation in the project communities, and the entire state, which made chemicals for farming highly valuable. A study on non-financial incentives for CHW in Ethiopia indicated that young age and being married were significant factors associated with motivation of the volunteers [23]. The majority of the CORPs were young or middle-aged, married men, many of whom were engaged with farming. In addition, the few women CORPs, needed and appreciated support for domestic chores, which they may not have had the time to do adequately while they volunteered as CORPs.

While it appeared reasonable and important for the community leaders and members in project communities to mobilise resources to support iCCM and the CORPs, some communities found the need more compelling than others. In LGAs where the resources mobilised were much lower, the communities and leaders were less responsive to advocacy and mobilisation activities.

### Retention of CORPs in iCCM

Nearly four-fifths of CORPs were active at the end of three years on the RAcE iCCM project, which was impressive, and higher than many other volunteer CHW programmes [24]. The self-esteem of CORPs was shown to be boosted, as community members showed their appreciation and commonly referred to them as “local doctors”, in recognition of their services. Although there are few quantitative studies on attrition or retention of volunteer CHWs, other authors have reported the proportion of volunteers remaining in active service as between 43.0% and 74.42% over a period of one to four years, in different community-based programmes [24]. In a recent study, a high attrition rate of 46.8/1000 person years was reported among CHWs in a maternal and child health project in Kenya [25]. Given this study was conducted retrospectively, it was beyond the study scope to learn more from CORPs about their reasons for leaving as none of the one-fifth of the trained CORPs that dropped out of the project was available to be interviewed. However, reports from community members showed that attritions were mainly due to relocations because of marriage, education, search for better livelihood and also because of death. Other authors have documented reasons why volunteer CHW were inactive or dropped out of service, including inadequate and irregular pay, lack of family support, receiving no feedback from supervisors, age, poor selection, absence of refresher training, and economic opportunities [25-27]. In this study we did not correlate levels of CORP retention directly with levels of community resources mobilised for their support. This is because there are many other factors, which were beyond the scope of this study to measure, which could have affected the levels of retention and thereby confounded any attempt to demonstrate causality of resource mobilisation on CORP retention. For example, the resources mobilised in Edati LGA were the highest in monetary value, yet the LGA had the lowest retention of CORPs. Other possible reasons for this high attrition rate may include more education or employment opportunities in other locations for example. It is important to note that this study also did not explore other possible contributing motivating factors for CORP retention including an individual's work-related goals, his/her sense of altruism or self-efficacy, job satisfaction, community valuation of CHW work, and fulfilment of pre-hire expectations among others [13].

### Resource mobilisation and sustainability

Two LGAs, Lapai and Rijau had proportionally more CORPs still active at the end of the project implementation than the others, and it was observed by the project team that the community and religious leaders in both were highly supportive of CORPs and actively promoted iCCM. In Lapai LGA, where about one-third of the total community resources were mobilised, there was a very high level of support to iCCM by the traditional and religious leaders. The open demonstration and declaration of support for the CORPs by the community leaders in some of the LGAs was observed as an impetus for the communities to provide resources for their CORPs, mainly farm produce and animal husbandry. Further, Rijau LGA has many very hard-to-reach areas in terms of terrain, and many communities in the LGA were completely cut-off from health facility for nearly 6 months’ during the rainy season every year. This could imply that it was an important priority for the communities facing a daily challenge of lack of health facilities and inadequate professional health workers, to provide the necessary support to the service of the CORPs. This fits with another study on hard-to-reach communities in a district in Kenya, where community and health system support are identified as crucial factors in sustaining and prolonging the service of CHWs [28].

In order to catalyse the duplication of success stories across project communities, best practices were shared at monthly LGA review meetings which encouraged other communities to replicate achievements. This was reported to have stimulated healthy competition among several communities who will not want to be seen as left behind.

In Niger state and in many parts of Nigeria, traditional and religious leaders are highly regarded as the “gatekeepers” of community norms. The public acknowledgment and appreciation of the importance of the work of the CORPs provided by the paramount traditional leader and popular religious leader of Lapai were a source of motivation for community members to mobilise resources for the CORPs.

The social bond between the memberships of the several benefitting communities has improved as a result of the iCCM services. Some community members would not attend meetings called by their leaders previously, however with the advent of iCCM community bonds have been strengthened. With the establishment and re-activation of the Village Health Committees (VHCs) and the Ward Health Development Committees (WHDCs) community responses to health issues have been strengthened. In addition to the WHDCs taking up projects such as the renovation of dilapidated Health Care centres and building new ones, the community members are now transformed advocates of iCCM, placing demand on the government to sustain iCCM.

The sustainability of iCCM is hinged on the resources dedicated to it. The effects of engagement and resource mobilisation which were observed by the project team have actual or potential positive effects on the sustainability of iCCM. The boosted self-esteem of CORPs may have made some difference in the motivation and aspirations of the CORPs. Self-esteem is one of the crucial factors that determines the job satisfaction or otherwise of a worker, which is related to remaining in-post and motivated to doing good work [10,29,30]. The election of CORPs as Ward Councillors can be motivating for existing CORPs or other CHWs, to be more dedicated to iCCM service, with the hope of a reward of traditional or political position in future. The healthy competition among communities, which resulted in a dedicated farm for iCCM, and the levy of amount considered affordable, are signs of community ownership of iCCM, which, if encouraged, will enhance sustainability of the project. The possibility of stoppage of donation and compensation is also a reason for the monthly levies, or dedicated farmland to plant crops and make sales, as communal effort to support iCCM.

Beyond the levy and dedicated farmland, the state government is also developing plans to harmonise and further institutionalise community health delivery services by adopting the Community Health Influencers, Promoters and Services (CHIPS) programme. The CHIPS programme was recently initiated by the National Primary Health Care Development Agency (NPHCDA) and recommended to state governments, as an approach to help institutionalize all CHWs, as integral to strengthening community health services. As part of CHIPS, all CHWs, irrespective of the content and type of their project, will have same designation; their selection, training, supervision, and compensation standardised and harmonized; as it is for other primary health care workers. Hopefully, when the CHIPS programme is operational, and the CORPs are absorbed into it, many of the issues related to drop-out of CHWs will be addressed.

### Study limitations and implications for future research

This study was a descriptive one and was not designed to present statistically significant data. Other limitations include the estimation of the in-kind, labour and gifts provided in support of the CORPs as a potential source of bias. The local resources mobilised for RAcE project were estimated based on the current market value of the in-kind contribution of community resources, as confirmed by the trade and market associations. For this study, the validation of the CORPs in active iCCM service was done in the third year of the project. Some trained CORPs have been active but were not available at the period of the validation, while others who were not usually available, just happened to be available by chance at the time the validation was done. Future research needs to consider assessment of the community resources mobilised and attrition of CHWs on more regular interval of one to two years to enable trend analyses to inform programme review. It is important to also conduct correlation of CORPs retention with levels of community resources mobilised for in-depth analysis of the factors that have implications for policy and service delivery. This is in view of the vital contribution of the CHWs to the initiatives towards universal health coverage.

## CONCLUSION

Mobilising local resources to support the RAcE project was informed by the need to motivate and support the CORPs, as identified in series of community dialogues. The level of community support in both monetary and non-monetary forms was high and appeared linked to the engagement and support of community leaders and mobilisers. Overall, the findings show the acceptability of iCCM in benefiting communities, and their willingness to contribute to uninterrupted service by CORPs to enhance sustainability of the project.

The achievements of community engagement, mobilisation, and the resources leveraged demonstrates the acceptability of the project by benefiting communities and their willingness to contribute to uninterrupted service provision by CORPs.
